# Severe child form of primary hyperoxaluria type 2 - a case report revealing consequence of GRHPR deficiency on metabolism

**DOI:** 10.1186/s12881-017-0421-8

**Published:** 2017-05-31

**Authors:** Jana Konkoľová, Ján Chandoga, Juraj Kováčik, Marcel Repiský, Veronika Kramarová, Ivana Paučinová, Daniel Böhmer

**Affiliations:** 1 Institute of Medical Biology, Genetics and Clinical Genetics, Comenius University, Faculty of Medicine & University Hospital Bratislava, Sasinkova 4, 811 08 Bratislava, Slovakia; 2 Department of Molecular and Biochemical Genetics – Centre of Rare Genetic Diseases, Faculty of Medicine & University Hospital Bratislava, Mickiewiczova 13, 813 69 Bratislava, Slovakia; 3 Department of Paediatrics, University Hospital Žilina, Vojtecha Spanyola 43, 012 07 Žilina, Slovakia

**Keywords:** Primary hyperoxaluria type 2, GRHPR, Oxalate, Glyoxylate reductase, Hydroxypyruvate reductase

## Abstract

**Background:**

Primary hyperoxaluria type 2 is a rare monogenic disorder inherited in an autosomal recessive pattern. It results from the absence of the enzyme glyoxylate reductase/hydroxypyruvate reductase (GRHPR). As a consequence of deficient enzyme activity, excessive amounts of oxalate and L-glycerate are excreted in the urine, and are a source for the formation of calcium oxalate stones that result in recurrent nephrolithiasis and less frequently nephrocalcinosis.

**Case presentation:**

We report a case of a 10-month-old patient diagnosed with urolithiasis. Screening of inborn errors of metabolism, including the performance of GC/MS urine organic acid profiling and HPLC amino acid profiling, showed abnormalities, which suggested deficiency of GRHPR enzyme. Additional metabolic disturbances observed in the patient led us to seek other genetic determinants and the elucidation of these findings. Besides the elevated excretion of 3-OH-butyrate, adipic acid, which are typical marks of ketosis, other metabolites such as 3-aminoisobutyric acid, 3-hydroxyisobutyric acid, 3-hydroxypropionic acid and 2-ethyl-3-hydroxypropionic acids were observed in increased amounts in the urine. Direct sequencing of the *GRHPR* gene revealed novel mutation, described for the first time in this article c.454dup (p.Thr152Asnfs*39) in homozygous form. The frequent nucleotide variants were found in *AGXT2* gene.

**Conclusions:**

The study presents metabolomic and molecular-genetic findings in a patient with PH2. Mutation analysis broadens the allelic spectrum of the *GRHPR* gene to include a novel c.454dup mutation that causes the truncation of the GRHPR protein and loss of its two functional domains. We also evaluated whether nucleotide variants in the *AGXT2* gene could influence the biochemical profile in PH2 and the overproduction of metabolites, especially in ketosis. We suppose that some metabolomic changes might be explained by the inhibition of the MMSADH enzyme by metabolites that increase as a consequence of GRHPR and AGXT2 enzyme deficiency. Several facts support an assumption that catabolic conditions in our patient could worsen the degree of hyperoxaluria and glyceric aciduria as a consequence of the elevated production of free amino acids and their intermediary products.

## Background

Primary hyperoxalurias (PHs) are a group of rare autosomal recessive inherited diseases. PHs are characterized by a defect in glyoxylate metabolism which results in endogenous oxalate overproduction. The clinical consequences of excessive oxalate excretion are nephrolithiasis and/or nephrocalcinosis, and/or the early-onset end-stage renal disease (ESRD) in childhood. Glyoxylate is a highly reactive metabolite that is normally effectively removed through its conversion to glycine and less effectively to glycolate. In humans, the glycine producing pathway is catalysed by the liver-specific peroxisomal enzyme alanine/glyoxylate aminotransferase (AGXT1; EC 2.6.1.44) also known as: serine-pyruvate aminotransferase (AGT1, AGXT, SPT, SPAT). The second metabolic pathway in which the glyoxylate is consumed by reduction to glycolate in the cytosol and mitochondria is catalysed by the enzyme glyoxylate reductase-hydroxypyruvate reductase (GRHPR; EC 1.1.1.79, alternative symbol: GLXR). In the case of the deficiency of one of the above-mentioned enzymes, a compensative mechanism takes over – the oxidation of glyoxylate to oxalate by cytosolic L-lactate dehydrogenase (LD). Also hydroxypyruvate that is normally reduced by GRHPR to D-glycerate is converted to L-glycerate by the same LD in the deficiency of enzyme GRHPR [1–4]. Under normal conditions, the competition between the reduction and oxidation of glyoxylate is balanced and controlled mainly by the cytoplasmic pool of NADPH, which can be utilized by GRHPR but not by LD and favours the production of glycolate, whereas LD activity favours the production of oxalate [5, 6]. Mutations in either the *AGXT1* or *GRHPR* gene that result in the synthesis of deficient proteins alter this equilibrium, and cause the overproduction of the main metabolites responsible for PHs. To date, the three types of PH (PH1, PH2 and PH3) have been described [7, 8]. Primary hyperoxaluria type 1 (PH1; OMIM #259900) is the most prevalent and most severe form of primary hyperoxaluria caused by AGXT1 deficiency. Primary hyperoxaluria type 2 (PH2; OMIM #260000 also known as L-glyceric aciduria) is less common than PH1 (exact incidence is unknown), and is characterized by a GRHPR enzyme defect. Recently, a third type of primary hyperoxaluria (PH3; OMIM #613616) has been described that is caused by the deficiency of the mitochondrial enzyme 4-hydroxy-2-oxoglutarate aldolase (HOGA), the apical enzyme in the mitochondrial hydroxyproline catabolism. Under physiological conditions, the enzyme splits HOG into pyruvate and glyoxylate, the latter being subsequently oxidized by the cytosolic LD to oxalate [8, 9]. HOGA enzyme deficiency results in HOG accumulation, however, the mechanism by which this deficiency causes hyperoxaluria has not been elucidated in detail yet. The inhibitory effect of HOG on the GRHPR enzyme has been assumed by Riedel [10] with consequences similar to PH2. Our study focuses on the deficiency of the GRHPR enzyme that possesses glyoxylate reductase (GR), hydroxypyruvate reductase (HPR), and D-glycerate dehydrogenase activities (DGDH) [11–14], which is causative of PH2. This homodimeric enzyme consists of 328 amino acids per subunit and is encoded by the *GRHPR* gene, located in the centromeric region of chromosome 9 and contains nine exons spanning 9 kbp. Though GRHPR deficiency is very rare, the current mutation database includes about 30 different types of mutations in the human *GRPHR* gene [2, 3, 15–17]. We report the case of a 10 month-old female patient with a clinical finding of urolithiasis who a clinician suspected of having a genetic disorder. Results of specific biochemical analyses and genetic examination led to the diagnosis of PH2 and the disclosure of a novel mutation in the *GRHPR* gene. Given the unexpected and unclear biochemical findings in relation to PH2, we subsequently sought genetic variants in the relevant gene - *AGXT2*, the protein of which is functionally closely coupled to GRHPR. Finally we evaluate all the important biochemical changes and genetic data observed in the patient, and discuss the possible metabolic consequences of GRPHR deficiency.

## Case presentation

A 10-month-old girl with a history of tonsilopharyngitis, bilateral inborn hip dysplasia with improvement, dispenzarised by an orthopaedist, was referred to hospital because of a one-day febrilities and positive urinary finding of ketone bodies, proteins, leukocyturia and haematuria. She was born after a normal pregnancy at full term with an uncomplicated perinatal and neonatal course. There was no history of gross haematuria or colicky abdominal pain, and she had received no medication except for antipyretics. The family history was negative with respect to renal and metabolic disease, including urolithiasis and nephrocalcinosis, however, genetic counselling revealed that the parents have common ancestors in the fourth generation.

**Fig. 1:**
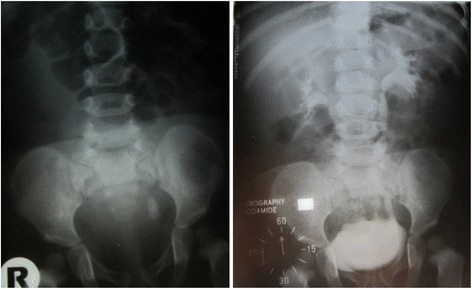
X-ray findings of urolithiasis in patient with PH2

On physical examination the child appeared mildly dehydrated with increased body temperature of 37.6 °C. A laboratory examination found moderate anaemia (haemoglobin 93.0 g/l), leucocytosis (15x10^9^/l) and moderately elevated CRP (42.6 mg/l). Serum electrolyte, urea and creatinine levels were normal. The calcium/creatinine ratio and 24 h calcium excretion was within the reference range. Blood gas analysis was normal with no evidence of metabolic acidosis. Ultrasonography of the abdomen raised suspicion of ureterolithiasis l.sin. Cystourethrography was indicated, showing concrement sized 2 cm x 0.8 cm in the distal part of the left ureter with mild dilatation of the renal pelvis and ureter (Fig. 1).

At the age of 11 months, an ureterolithotomy was performed at the University Children Hospital Bratislava. A chemical analysis of the extracted stone was performed, and the stone was characterised as a composite of virtually pure calcium oxalate. The following course was without any complications.

## Biochemical analyses

### Methods

Biochemical analyses were performed in the Department of Molecular and Biochemical Genetics of University Hospital, and the obligatory recommended schemes and flow charts were used to elucidate possible metabolic disorders. Crucial for the diagnostics of rare monogenic forms of urolithiasis is the application of chromatographic methods such as HPLC, GC/MS focused on amino acids, and the organic acids profile of urine.

### Determination of amino acids in urine

Forty nmol of norvaline was added as an internal standard to each deproteinised urine sample. The routine HPLC procedure recommended by Agilent Technologies was applied for the separation of amino acids using the Zorbax Eclipse AAA HPLC column and on line derivatisation with OPA-MPA. For the analysis, a HPLC instrument of Agilent Technologies 1200 series was used with the diode array detector operating at 338 nm. The calculation of amino acid concentration was performed in relation to areas of internal standard in the sample.

### Determination of organic acids in urine

The determination of organic acids in the urine sample was performed by gas chromatography/mass spectrometry (GC/MS) using an ITQ 1100 Thermo Scientific ion trap mass spectrometer operating in split mode (split ratio 1:40). The separation of urine metabolites was achieved on 30 m DB5 column with 0.25 i.d. (Agilent) and constant helium column flow 1.2 ml/min during 28 min. Equal aliquot of the internal standard (0.61 mmol of 4-phenylbutyric acid) was added to 1 ml - 2 ml of urine sample, and organic acids were extracted by ethylacetate. The derivatisation of organic acids was performed by a mixture of BSTFA/DMCS/acetonitrile/pyridine (10:5:1:1) in a heating block for 60 min at 60 °C. For the calculation of excreted urine compounds, the calibration curves were constructed from four different points of concentrations of standards. All measured values were corrected on the recovery of added internal standard. The urinary excretion of organic acids was expressed in mmol/mol creatinine. The values of all measured metabolites in the patient’s urine were compared with the reference data published by Blau et al., 2008 and the Urine Metabolome Database www.urinemetabolome.ca.

### Results

The analysis of amino acids in urine is part of the routine scheme focused on the diagnosis of defects in amino acid metabolism or tubular transport. In the urine sample we found only mildly elevated lysine and cystine excretion, while the excretion of arginine and ornithine was within reference values. We also found elevated excretion of β-aminoisobutyric acid (BAIB) - 137 mmol/mol creatinine (laboratory control ranges are 9–45 mmol/mol creatinine). Nevertheless, such finding is frequent in the Caucasian population and is caused by the presence of single nucleotide variants in the *AGXT2* gene, upon which genetic analysis was additionally performed.

**Fig. 2:**
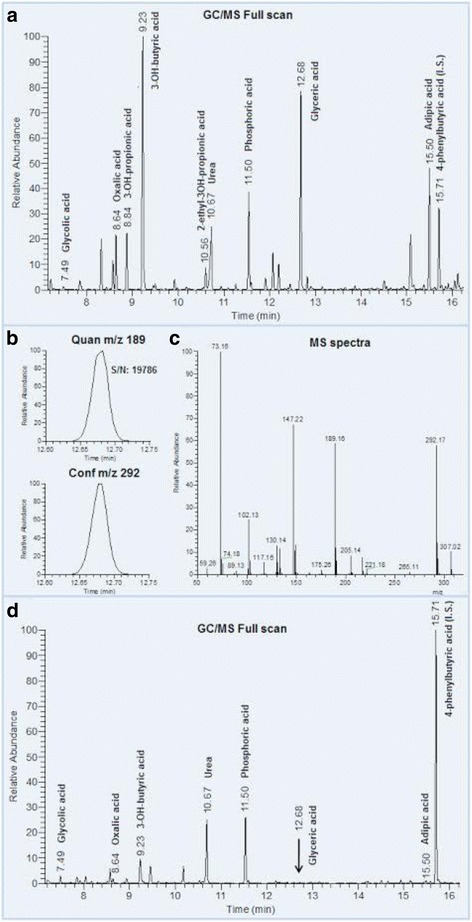
GC/MS chromatogram of urine organic acids of patient with PH2 and control sample. **a** The total ion current chromatogram of urine organic acids of patient sample with markedly elevated peak in retention time 12.68 corresponding to glyceric acid (MU-1342). **b** The presence of two characteristic ions for glyceric acid with high signal to noise ratio (S/N) for quantitative ion in patient’s sample. **c** MS spectra of peak corresponding to glyceric acid. **d** The total ion current chromatograph of urine organic acids of control sample with negligible peak of glyceric acids

A routine GC/MS analysis of organic acids in the patient’s urine samples revealed a marked peak in/with a retention time of 12.7 min corresponding to compounds with retention index 1342 MU (methylene units). On the physiological urine chromatogram this peak is negligible. A comparison of the obtained chromatographic data with the library mass spectra (NIST library) revealed that the peak corresponded to glycerate (Fig. 2). Quantification of organic acids content in the urine uncovered clear-cut abnormalities when compared to accepted refference values.Oxalate was only moderately elevated up to 349 mmol/mol creatinine (reference age-related interval 61–162 mmol/mol creatinine). In contrast to the oxalate, the glycerate value was extraordinarily high, reaching 2796 mmol/mol creatinine (reference only trace). On the other hand, the glycolate urine excretion was normal, reaching 69 mmol/mol creatinine (reference interval 22–139 mmol/mol creatinine). The finding of extremely high glycerate excretion was crucial for the choice of gene responsible for methabolic pathology and molecular genetic diagnostic. In addition to this principal metabolic finding, we found increased excretion of 3-hydroxybutyrate (HBA; 605 mmol/mol creatinine), 3-hydroxyisobutyric acid (HIBA; 80.2 mmol/mol creatinine), and adipate (134 mmol/mol) in the examined urine sample - all typical marks of ketosis. In comparison to the control samples, two trace metabolites 3-hydroxypropionic acid (HPA, alternative name hydracrylate) and 2-ethyl-3-hydroxypropionic acids (EHPA; alternative names: 2-hydroxymethyl-butyrate, 2-ethylhydracrylate) were also substantially elevated in the urine. The quantity of HPA was estimated by an obligatory procedure based on the comparison of ion areas (Q-177) of the patient's sample, and HPA standard and revealed value 593 mmol/mol creatinine. Due to the absence of a commercially available standard, EHPA quantification was performed using arbitrary units (ratio of metabolite area to area of standard). According to data from the Human Urine Metabolome Database, the average value of EHPA is 4 mmol/mol creatinine, and based on this finding and data from our control samples we estimate that the excretion of EHPA may be approximately 40 mmol/mol of creatinine in the patient’s urine sample. In the chromatogram we observed an unknown metabolite at a retention time corresponding to 1500 MU.

The measurement of urine organic acids level in the patient’s second sample (five days after surgical intervention) confirmed increased values of oxalate and glycerate (167 mmol/mol and 1113 mmol/mol creatinine respectively), however, normal values of HBA and adipate were observed. Furthermore, the excretion of BAIB acid returned to the physiological level (27 mmol/mol creatinine). As for HPA and EHPA, while the EHPA levels decreased to normal, the excretion of HPA remained significantly elevated. The unknown metabolite observed in the first sample was not detected in this urine sample. In order to interpret the observed secondary metabolic changes correctly, we decided to compare the increase in the urine excretion levels of all the mentioned metabolites with the control samples and the samples of ketonuric patients obtained from our database (Table 1). We used values of 11 control samples of probands of both genders and aged more than 6 months.

**Table 1:** The comparison of urine excretion of metabolites related to primary hyperoxaluria and ketosis in patient and control urine samples with/without ketosis. Values are expressed in mmol/mol creatinine as means ± standard deviation (BAIB - β-aminoisobutyric acid, HBA - 3-hydroxybutyrate; HIBA - 3-hydroxyisobutyric acid; HPA - 3-hydroxypropionic acid; EHPA - 2-ethyl-3-hydroxypropionic acids)

Metabolite	Control samples with ketosis (*n* = 11)	Control samples without ketosis (*n* = 11)	Patient with ketosis	Patient without ketosis
Glycerate	1.54 ± 1.34	0.97 ± 0.67	2796	1113
Oxalate	29.1 ± 17.1	37.1 ± 19.2	349	167
Glycolate	92.1 ± 62.3	51.3 ± 26.7	69	106
HBA	1890 ± 2481	5.1 ± 6.6	605	2.1
HIBA	20.0 ± 16.7	10.6 ± 5.5	80	29.5
HPA	28.9 ± 35.2	3.9 ± 2.4	593	132
EHPA	13.9 ± 13.6	4.0 ± 1.5	38.2	6.1
BAIB	54.2 ± 33.5	15.7 ± 8.1	137	27

## Molecular identification of the mutations

### Methods

#### DNA isolation and PCR reactions

Genomic DNA was isolated from whole blood samples using the NucleoSpin Blood Kit (Macherey-Nagel). The DNA encoding of the *GRHPR* gene (ENST00000318158) was amplified using primers as described in Table 2. Primer sequences were based on the published genomic DNA sequence accession number AF146689. All nine PCR fragments were performed in 20 μl volumes containing approximately 100 ng of DNA, 2x PCR Master Mix (Thermo Fisher Scientific), and 0.5 μmol/l of each primer. The PCR program for all regions was as follows: 3 min at 95 °C, and then 40 cycles at 95 °C for 30s, 55 °C for 30s, and 72 °C for 30s, followed by 7 min at 72 °C for the final extension.

**Table 2:** Primers used for amplification of genomic *GRHPR* DNA

Name	Primer sequence (5’ → 3’)
GRHPR-1F	GCCAGCTTCTGTACTGCCA
GRHPR-1R	CTCCGAGACTCCCCAAAACT
GRHPR-2F	GACAGGTGTGCGGCTCCT
GRHPR-2R	CAAGCCACCCTCAAGTCC
GRHPR-3F	GCTGTGGCTTTGAGTTCCTC
GRHPR-3R	GCCGAGGGATATGCAGTAAA
GRHPR-4F	GCAGATCAAAGAGGGAGCAA
GRHPR-4R	CACCTGGTCTGCGTTCACT
GRHPR-5F	TTGGACCACAGTCAGAGGTG
GRHPR-5R	GCCAGGGATGCAAACCA
GRHPR-6F	GAAAAGGGTCTGCCCTGAG
GRHPR-6R	CAACTGGGCACAGATAGGC
GRHPR-7F	CCATCTGGTTGTCCCTAGCC
GRHPR-7R	CTCCAGGCTTGCTGGGTA
GRHPR-8F	GGAGGGATCTTCGGGGTA
GRHPR-8R	ACCCCCTCAAAAACACTGGT
GRHPR-9F	CAGCTGAAGGCTGCTGAAC
GRHPR-9R	AGAATCACACCTTCCCTTGG

#### Sequencing analysis

The sequencing analysis of PCR fragments was performed after their purification using Exonuclease I and FastAP (Thermo Fisher Scientific). Both sense and antisense strands were sequenced with a DNA-sequencing kit - Big Dye terminator cycle sequencing ready reaction version 3.1 (Applied Biosystems) – and subsequently purified using the Nucleospin column purification system (QIAGEN). The electrophoresis of amplified products was performed using an ABI PRISM®3100 (Applied Biosystems), with 50 cm capillary columns loaded with Alter-POP6 polymer (Applied Biosystems). Analytic data was treated using SeqScape v2.6.0. software.

### Results

**Fig. 3:**
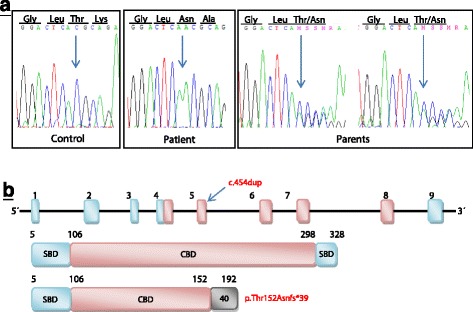
The structure of *GRHPR* gene and protein and sequences with mutation c.454dup. **a** Sequences show a control samples without mutation and a duplication of nucleotide A in homozygous (patient) and heterozygous state, which changes the followed amino acids from origin threonine to asparagine. The last two sequences are the parents of the patient. **b** *Arrow* shows the localization of this mutation in gene *GRHPR* (in exon 5) and diagram shows a predicted shorted protein. *Boxes* indicate exons and lines indicate introns. The coenzyme-binding domain (CBD) is indicated by a *red box* and two the formate/glycerate dehydrogenase substrate-binding domain (SBD) are indicated by *blue boxes*. The *grey box* indicates the length of additional amino acids that is appended as a result of the frame-shifting mutations

PCR amplification of genomic DNA followed by the sequencing of all nine exons and the intron-exon boundaries in the *GRHPR* gene (NM_012203.1) revealed several nucleotide variants. Homozygous synonymous variant c.579A > G (p.Ala193=, rs309458) and homozygous variant c.288-11C > T (rs2736664) were found in the exon 6 and intron 3, respectively. In the exon 5, we identified mutation c.454dup in homozygous form (Fig. 3a). This causal mutation has not been reported before [44] and results in a frameshift (p.Thr152Asnfs*39) after the insertion of a single A nucleotide. The frameshift generates a truncation of the GRHPR protein - from the original 328 amino acids to a 192-amino-acid-long polypeptide. In the truncated protein, the first 152 amino acids remain the same as in the wild-type protein, whereas the terminal 40 amino acids display an altered sequence. The frameshift causes the loss of two essential functional domains – the coenzyme-binding domain (CBD) and the formate/glycerate dehydrogenase substrate-binding domain (SBD). To get a full picture, we also analysed the DNA of both the patient’s parents. Sequence analysis of the *GRHPR* gene showed that both were heterozygous for the causal c.454dup mutation.

We performed a genetic analysis of the *AGXT2* gene (ENST00000231420) coding the AGXT2 enzyme (alternative names: Alanine Glyoxylate Aminotransferase 2; Beta-Alanine-Pyruvate Aminotransferase; AGT2; DAIBAT) (E.C. 2.6.1.40), and we found the five nucleotides variants. The two nucleotides variants in homozygous state, rs37370, (serine 102 asparagine) and rs180749, (threonine 212 isoleucine) were referred as responsible for the loss of AGXT2 activity and increased BAIB excretion [18, 19].

## Discussion and conclusions

Using a routine HPLC analysis of urinary amino acids, we detected only a mildly elevated excretion of lysine and cystine in the patient’s sample, and hence we excluded cystinuria as a cause of the diagnosed urolithiasis. Cystinuria (OMIM #220100) is a frequent disease inherited in an autosomal recessive fashion that is caused by the insufficient transport of cystine and basic amino acids (lysine, ornithine, and arginine) through renal tubules and enterocytes. Although the manifestation of cystinic urolithiasis in children is rare, it was necessary to exclude this defect as the cause of the disease.

The determination of organic acids and certain glycine conjugates in urine by GC/MS is a widespread approach used in the diagnosis of inherited metabolic disorders, including the monogenic forms of urolithiasis. However, the procedure consisting of liquid extraction, derivatisation, and chromatographic separation of organic acids bears non-negligible limitations. Furthermore, the excretion of metabolites in the pathological condition is variable and, among other factors, may be influenced by the type of mutation, dietary regime, fasting and therapy. The limitations of the routinely applied methods affect the diagnostics of primary hyperoxalurias significantly, and hence may obscure the published statistical data concerning the incidence and prevalence of the disease. Only two cases of PH1 have been described in Slovakia, and the presented case of PH2 is the first report of the disease in our country. Coupled chromatographic and mass spectrometric analysis resulted in the identification of glyceric acid as a pathognomonic compound in the patient’s urine samples. When we then analysed several control urine samples, we found only traces of glycerate in them. An ineffective extraction procedure employing ethyl acetate could explain the observed low recovery of many polar compounds, including glycerate, in urine. Although novel effective methods for the extraction of polar compounds such as oxalate, glycolate, glycerate have been introduced [20], they have not been used for routine analysis yet. Another limitation of the routinely applied GC/MS methods is the inability to distinguish between two optical isomers. Special columns with chiral stationary phase or utilization of chiral reagents, in combination with ordinary non-chiral column [21] in the diagnosis of PH2 characterised by the occurrence of L-glycerate, could alleviate this limitation. A common peak for the two optical isomers in the GC/MS analysis is a key problem in the evaluation of glyceric aciduria type. The two D(+) and L(−) glyceric acids enantiomers are normally excreted in trace amounts in urine [22], however, both are important biochemical markers of two rare inherited metabolic diseases with different phenotypes. The first disease is the D-glyceric aciduria characterized by D-glycerate kinase (*GLYCTK* gene) enzyme deficiency, whereas the second disease, PH2, is accompanied by L-glyceric aciduria and hyperoxaluria caused by a deficiency of the GRHPR enzyme. D-glyceric aciduria is a very rare disease with variable phenotype; however, neurological and developmental manifestations predominate. The clinical and biochemical findings in our patient were not relevant to this diagnosis. The presence of a clinical picture of ureterolithiasis proved by X-ray was highly suggestive of the diagnosis primary hyperoxaluria. Whereas imaging methods and chemical analysis of the urine stone could not reliably distinguish between PH1 and PH2, the applied GC/MS method with principal finding of high glycerate and oxalate versus normal glycolate concentrations in the urine helped us to resolve the diagnosis correctly. However, we have to stress the fact that just an extremely high excretion of glycerate was substantial in the biochemical diagnostics of this disease in the patient.

Fasting and the resulting ketogenesis accompanied by gluconeogenesis have undoubtedly worsened the biochemical manifestations of enzyme deficiency in the patient, this due to the increased serine and hydroxyproline flux and the subsequent overproduction of hydroxypyruvate and glyoxylate. Based on the molecular-genetic findings and enzyme studies, it is assumed that PH2 could manifest without hyperglyceric aciduria, as was referred by Rumbsy et al. [23]. Hence molecular-genetic analysis has to be favoured as crucial proof of the presence or absence of the disease. It is recommended to seek the mutation in *AGXT1* in the first step, and in the analysis of *GRHPR* and *HOGA1* genes in the second step when PH is suspected and the biochemical phenotype is not clear-cut expressed.

The assumption of PH2 was unequivocally confirmed by the molecular-genetic analysis of the *GRHPR* gene, which disclosed the presence of homozygous mutation c.454dup in the patient and the same mutation in heterozygous state in her parents. This homozygous form of mutation causes the frame shift after amino acid 152, which is followed by 40 novel amino acids and a premature stop codon at amino acid position 192. The generated protein has only 192 amino acids in comparison to the original 328 amino acids. The lacking of 136 amino acids is responsible for the loss of the important coenzyme-binding domain (CBD) of protein in position 107 to 298, and as well as the second domain of the two formate/glycerate dehydrogenase substrate-binding domain (SBD) located in the region 299–328 amino acids (Fig. 3b.) [24, 25].

**Fig. 4:**
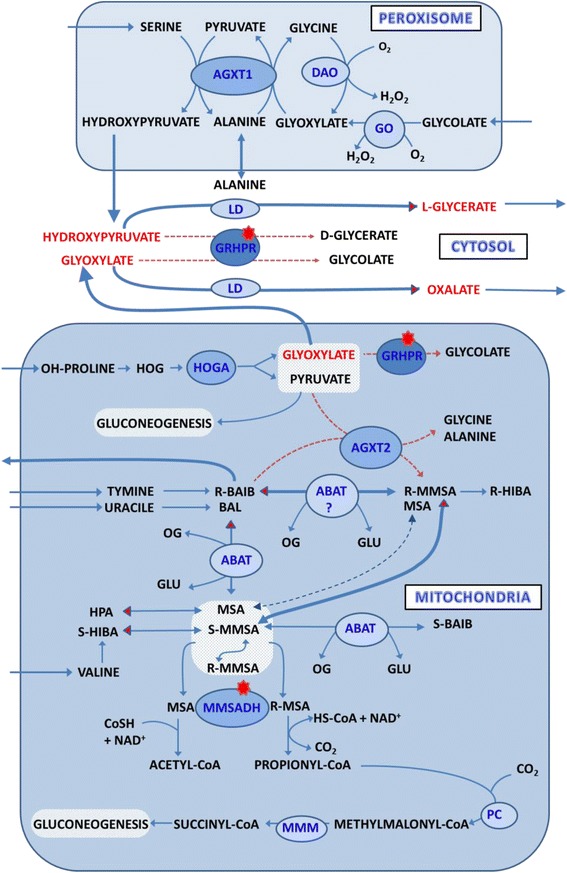
The scheme showed the probably metabolic pathways in patient with PH2 and AGXT2 deficiency. The *red dots* indicate deficient enzymes, *red dashed arrows* point to the deficiency of metabolic pathway, thick *blue arrows* represent the overproduction of enzymes and *red* ends of the *blue arrows* point to the overproduced metabolite. Abbreviations: DAO – D-aminoacid oxidase; AGXT1 – alanine-glyoxylate aminotransferase1; GO – glycolate oxidase; LD – lactate dehydrogenase; HOGA - 4-hydroxy-2-oxoglutarate aldolase; AGXT2 – alanine-glyoxylate aminotransferase 2; ABAT – aminobutyrate-2-oxoglutarate; MMSADH – methylmalonyl semialdehyde dehydrogenase; MMM – methylmalonyl-CoA mutase; GRHPR - glyoxylate reductase-hydroxypyruvate reductase; PC – propionylcarboxylase

During the attack coupled with febrilities and ketosis, additional metabolic disturbances were observed in the patient which led us to seek other genetic determinants and the elucidation of these findings. Since low amounts of glycolic acid were present in the urine of the patient, we suggest that the AGXT1 enzyme correctly catalyses the conversion of glyoxylate to glycine, and that the detoxification of this harmful metabolite in peroxisomes is effective. The production of glyoxylate in mitochondria by the HOGA enzyme and its detoxification in this compartment is less satisfactorily explained. Based on known data, it can be inferred that except for the GRHPR enzyme, mitochondrial AGXT2 can effectively use pyruvate and glyoxylate also for the transamination of D-3-aminoisobutyrate (D-BAIB) and β-alanine (BAL) [26]. It has been long established that genetic factors influence the high excretion of BAIB, originated preferentially from the degradation of thymine (R- form) and to a lesser extent from valine (S-form) [27–30]. In the patient’s urine we observed an elevated excretion of BAIB, which is usually related to the diminished activities of AGXT2 enzyme. This frequent finding in Caucasians is typically caused by the presence of the five nucleotides variants. In our patient two such variants were found in homozygous state, rs37370 and rs180749, and were referred to in literature as responsible for the loss of AGXT2 activity and increased urinary (BAIB) excretion [18, 19]. According to Landaans and Solen [31], the excretion amounts of this amino acid in urine (95% in the form of R isomer) increased markedly in patients with ketoacidosis, a phenomenon that was also observed in our patient. An important finding was reported by Gennip et al. [32] that urine has a constant ratio between R and S isomers (20:1), supporting the idea that interconversion between enantiomers can occur. It is unequivocally accepted that D-BAIB is generated by the conversion of valine metabolite S-methylmalonyl semialdehyde (S-MMSA) to R- methylmalonyl semialdehyde (R-MMSA), and in the second step to R-BAIB by transamination. A critical point in this claim is transamination because of the stereospecificity of the supposed enzyme ABAT (E.C.2.6.1.19). This enzyme catalyses the transamination of non-stereospecific BAL, GABA and from stereospecific isomers only L-BAIB with 2-oxoglutarate [33, 34]. In a report subsequently published by Tamaki [26], a mixture of appropriate substrates and enzymes AGXT2 and ABAT provide in vitro condition conversion between S-BAIB and R-BAIB. In context with genetic abnormality (AGXT2 deficiency), we consider changes of the mitochondrial pool of HOGA, glyoxylate, and their additive inhibitory effects on GRHPR that accentuate deficiency. Except for the basic biochemical finding related to HP2, the GC/MS analysis of urinary organic acids disclosed an increase in HBA and adipic acid excretion. These observations are in accordance with the health status of our patient with food restrictions that resulted in the increase of ketone bodies and dicarboxylic acids production. In addition, other minor metabolites (HIBA, HPA, EHPA, 2-methyl-3-hydroxybutyrate) were found in elevated quantities in the urine of patients with ketoacidosis [35, 36]. When we compared the excretion of these metabolites in our patient’s urine with control urine samples (to means of values), the excretion of HIBA was elevated 8 times, EHPA 10 times, and HPA even 150 times. Subsequently when we compared the patient’s values with those of patients with ketosis, the elevation for HIBA is 4 times EHPA 3 times, and HPA 20 times. Data regarding the correlation between HBA and other mentioned metabolites in urine from patients with ketoacidosis has been very rare [35]. In our cohort of patients with ketosis, we observed a relationship between HBA and HIBA excretion, but not between HBA and HPA or EHPA. The discrepancy in the degree of excretion of ketone bodies and studied metabolites is apparent at least for HPA in our patient. A possible explanation is that there is an influence of overproduced metabolites such as HOGA and glyoxylate on routes producing these trace metabolites, probably by inhibitory mechanism. Catabolic conditions and amino acid break down probably augment this effect. Inspiring for the assessment of this thesis was data published by Pollitt et al. [37], who reported a child with methylmalonyl semialdehyde dehydrogenase deficiency (MMSADH - E.C. 1.2.1.27). Biochemical findings were characterised by an excessive excretion of amino acids R-BAIB, S-BAIB, BAL, and hydroxyacids such as HPA, S-HIBA and EHPA, and were logical consequences of MMSADH deficiency. Metabolic disorders in the child were confirmed by the finding of a homozygous mutation 1336G > A (Gly446Arg) by Chambliss et al. [38]. Catalytical properties of a rat liver mitochondrial enzyme isolated by Goodwin et al. [39] enable NAD^+^ dependent dehydrogenation of malonate semialdehyde (MSA) and MMSA. This enzyme participates in the metabolism of valine, thymine, uracile, and their catabolic products such as BAL and both isomers of BAIB. In relation to the metabolism of MMSA kinetic data characterised by Godwin et al. [39], Kedishvili et al. [40] and interconversion studies published by Manning and Pollitt [41] indicate that both stereo isomers are used by MMSADH, though the R isomer is preferred. In our patient all the above-mentioned metabolites (BAL was not measured) show a significant increase in excretion, and are in thin connection with the function of MMSADH. A possible explanation is the inhibitory effect of overproduced metabolites such as HOGA or unconsumed products such as glyoxylate on MMSADH due to the deficiency of GRHPR and AGXT2. In regard to the close structural resemblance to MSA, we consider that glyoxylate may be favorised as the inhibitor of the enzyme. The net of metabolic changes in mitochondria, peroxisomes and cytosol of patient is depicted in Fig. 4.

In conclusion we consider that several facts support the assumption that catabolic conditions in PH2 patients could worsen the degree of hyperoxaluria and glyceric aciduria as a consequence of the elevated production of free amino acids and their intermediary products. This situation is triggered by urinary colic and leads to a vicious circle. Hence mainly in children it is reasonable as soon as possible to sustain parenteral energetic substitution. We would like to point out the importance of considering rare inborn errors of metabolism in differential diagnosis of urolithiasis, especially when manifested in young age.

## Abbreviations

AGXT: Alanine/glyoxylate aminotransferase
GC/MS: Gas chromatography/mass spectrometry
GR: Glyoxylate reductase
GRHPR: Glyoxylate reductase/hydroxypyruvate reductase
HPR: Hydroxypyruvate reductase
LD: L-lactate dehydrogenase
PH2: Primary hyperoxaluria type 2
